# Rapidly destructive coxarthrosis accompanied by fluctuating C‐reactive protein level

**DOI:** 10.1002/ccr3.5131

**Published:** 2021-11-25

**Authors:** Hayato Shimizu, Hiroaki Nishioka

**Affiliations:** ^1^ Department of General Internal Medicine Kobe City Medical Center General Hospital Kobe Japan

**Keywords:** C‐reactive protein, radiography, rapidly destructive coxarthrosis

## Abstract

Rapidly destructive coxarthrosis is a rare entity of unknown etiology that is characterized by rapid hip joint destruction. Blood tests are thought to be non‐specific. However, we herein show a patient with rapidly destructive coxarthrosis, which was accompanied by fluctuating C‐reactive protein level.

## Graphical abstract

Rapidly destructive coxarthrosis should be in the differential diagnosis for acute hip pain with an elevated CRP level, even if the joint deformity is slight on radiography, and early surgery should be considered.

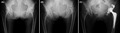

A 74‐year‐old woman presented with a 3‐day history of left hip pain. Her left hip joint exhibited tenderness, and pain was induced by rotation. A CRP level was elevated (11.83 mg/dl). Radiography of the left hip slightly demonstrated joint space narrowing (Figure 1A). Synovial fluid cultures of the left hip yielded no bacteria. Her pain deteriorated and elevated CRP level continued, but the radiographs did not show any obvious changes. Orthopedic surgeons hesitated an operation because of the high CRP level. Approximately 2 months after the presentation, radiography revealed marked destruction of the left femoral head (Figure 1B). We diagnosed her with rapidly destructive coxarthrosis (RDC). Left hip arthroplasty was performed (Figure 1C). Her pain disappeared, and CRP returned to normal level.

**FIGURE 1:**
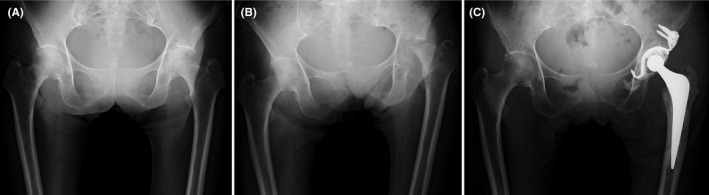
(A) Anteroposterior radiograph of the left hip showing joint space narrowing. (B) Radiograph shows complete destruction of the femoral head. (C) Radiograph shows left total hip replacement

Rapidly destructive coxarthrosis is a rare entity of unknown etiology. Initial presentation is acute hip pain without radiographic evidence of joint destruction. Complete vanishing of the proximal femur abruptly occurs within a few months.^1^, ^2^ Early surgery is desirable.^2^ Blood tests are thought to be non‐specific^1^; however, our patient showed that RDC can be accompanied by fluctuating CRP. This suggests that RDC should be in the differential diagnosis for acute hip pain with elevated CRP level, and early surgery should be considered.

## CONFLICT OF INTEREST

The authors declare that they have no competing interests.

## AUTHOR CONTRIBUTIONS

HS collected the data and wrote the first draft of the manuscript. HN coordinated the project and edited the manuscript. Both authors have read and approved the final manuscript.

## ETHICAL APPROVAL

Written informed consent was obtained from the patient. This case is anonymous.

## Data Availability

Data sharing is not applicable to this article as no datasets were generated or analyzed during the current study.
